# Antibacterial and Antibiofilm Activities of Chlorogenic Acid Against *Yersinia enterocolitica*

**DOI:** 10.3389/fmicb.2022.885092

**Published:** 2022-05-04

**Authors:** Kun Chen, Chuantao Peng, Fang Chi, Chundi Yu, Qingli Yang, Zhaojie Li

**Affiliations:** ^1^College of Food Science and Engineering, Qingdao Agricultural University, Qingdao, China; ^2^Qingdao Special Food Research Institute, Qingdao, China; ^3^CAS Key Laboratory of Biofuels, Qingdao Institute of Bioenergy and Bioprocess Technology, Chinese Academy of Sciences, Qingdao, China

**Keywords:** chlorogenic acid, antibiotic resistance, *Yersinia enterocolitica*, antibacterial activity, antibiofilm activity

## Abstract

Nowadays, developing new and natural compounds with antibacterial activities from plants has become a promising approach to solve antibiotic resistance of pathogenic bacteria. Chlorogenic acid (CA), as a kind of phenolic acid existing in many plants, has been found to process multifunctional activities including antibacterial activity. Herein, the antibacterial and antibiofilm activities of CA against *Yersinia enterocolitica* (*Y. enterocolitica*) were tested for the first time, and its mechanism of action was investigated. It was demonstrated that CA could exert outstanding antibacterial activity against *Y. enterocolitica*. Biofilm susceptibility assays further indicated that CA could inhibit biofilm formation and decrease the established biofilm biomass of *Y. enterocolitica*. It was deduced that through binding to *Y. enterocolitica*, CA destroyed the cell membrane, increased the membrane permeability, and led to bacterial cell damage. In addition, the transcriptomic analysis revealed that CA could disorder many physiological pathways, mainly including the ones of antagonizing biofilms and increasing cell membrane permeability. Finally, the spiked assay showed that the growth of *Y. enterocolitica* in milk was significantly inhibited by CA. Taken together, CA, as an effective bactericidal effector with application potential, exerts antagonistic activity against *Y. enterocolitica* by mainly intervening biofilm formation and membrane permeability-related physiological pathways.

## Introduction

*Yersinia enterocolitica* is a facultative anaerobic, gram-negative coccoid bacillus and a ubiquitous foodborne pathogen (Bottone, 2015), which is widely distributed in meat, vegetables, dairy, and aquatic products (Liang et al., 2019). This pathogen is mainly transmitted through food or water sources, causing a gastrointestinal disease in humans, also known as yersiniosis. Yersiniosis is the fourth most commonly reported bacterial foodborne zoonosis in Europe, which poses a great threat to the health and life of people worldwide (Efsa and Ecdc, 2018). The common symptoms of yersiniosis include diarrhea, abdominal ache, nausea, and vomiting (Wang et al., 2019a). *Y. enterocolitica* can proliferate at a lower temperature (4°), making it dangerous for refrigerated food (Leon-Velarde et al., 2019).

Biofilms are a non-negligible food safety concern in controlling hygiene in food industry. Microorganisms have a natural tendency to attach to abiotic and biotic surfaces, resulting in the formation of a biofilm (Borges et al., 2012), which is commonly associated with periodontal disease, cystic fibrosis, osteomyelitis, endocarditis, and infections related to surgical implants (Raja et al., 2011). Moreover, biofilms possess a typical characteristic of having extreme resistance to many physical and chemical factors, especially antibiotics (Borges et al., 2012). As many pathogenic bacteria like *Staphylococcus aureus* (*S. aureus*) and *Stenotrophomonas maltophilia* (*S. maltophilia*), *Y. enterocolitica* can also form biofilms which may be related to its pathogenicity, antibiotic resistance, and virulence (Di Marco et al., 2020).

In order to tackle these infectious diseases caused by pathogens, many antibiotics have been developed. However, the misuse or overuse of antibiotics can lead to increasing antibiotic resistance (AR) of pathogenic bacteria, which has become one of the greatest threats to public health (Qiao et al., 2018). Therefore, there is an urgent need to develop new and effective antibacterial compounds with novel modes of action or as alternatives to antibiotics to address these serious issues.

Naturally derived agents with antibacterial activity have been increasingly studied over the years. For example, many natural active compounds extracted from medicinal plants, such as coptisine (Wu et al., 2019), berberine (Peng et al., 2015), baicalin (Luo et al., 2017), and so on, have been shown to possess antibacterial activities. Some natural antimicrobial compounds like plant polyphenols can inhibit several different groups of biomolecules in a pathogen, making the development of resistance to such compounds unlikely (Harrison et al., 2008; Miller et al., 2009). Also, some natural antibacterial compounds can act on bacterial biofilms to inhibit the formation of biofilms or destroy the integrity of biofilms, finally killing bacteria (Lin et al., 2018; Li et al., 2019a).

Chlorogenic acid (CA) is one of the most important compounds with outstanding antibacterial activity. CA, a kind of phenolic acid from the hydroxycinnamic acid family, has been found in many plants and foods (Nabavi et al., 2017), such as *Eucommia ulmoides* (Huang et al., 2019), *Lonicera confuse* (Huang et al., 2019), *Prunus domestica* (Alsolmei et al., 2019), fruits, wine, olive oil, and coffee (Nabavi et al., 2017). CA possesses many pharmacological effects, including antibacterial, antioxidant, lipid-lowering, antiviral, anti-inflammatory, anti-cardiovascular, anticancer, and immunomodulatory effects (Miao and Xiang, 2020). So far, CA has been widely used in many fields such as medicine, food, health care, and the chemical industry. Regarding its antibacterial activity, CA had broad-spectrum antibacterial activities against many bacteria, such as *S. aureus* (Li et al., 2013), *Escherichia coli* (*E. coli*) (Li et al., 2006), *Pseudomonas aeruginosa* (*P. aeruginosa*) (Wang et al., 2019b), *S. maltophilia* (Zhang et al., 2019), *Bacillus subtilis* (*B. subtilis*) (Wu et al., 2020), yeast, and *Aspergillus Niger* (*A. Niger*) (Miao and Xiang, 2020). However, studies related to the antibacterial activity of CA against *Y. enterocolitica* are limited. Besides, the specific mechanism of action is still unclear.

In this study, we investigated the antibacterial and antibiofilm activities of CA on *Y. enterocolitica*. Furthermore, the mechanism of action was systematically studied through a set of experiments. This study will promote the application of CA in food, medicine, health care, and the chemical industry.

## Materials and Methods

### Bacterial Strains and Chemicals

*Yersinia enterocolitica* ATCC 23715 was purchased from the *American Type Culture Collection* (ATCC) and was activated in *Luria-Bertani* (LB) Nutrient Agar (*Hopebio*, Qingdao, China) for 24 h in a 37° constant temperature incubator. Before use, bacteria were cultured in LB broth (*Hopebio*, Qingdao, China) for 12 h with constant shaking (150 rpm) at 37°.

Chlorogenic acid (≥ 95%, CAS 327-97-9), extracted from *Eucommia ulmoides*, was purchased from *Sigma-Aldrich* (St. Louis, United States) and dissolved in sterile water for use. Crystal violet (CAS 548-62-9) was obtained from *Solarbio* (*Solarbio* Science & Technology Co. Ltd, Beijing, China). L7012 *LIVE/DEAD BacLight Bacterial Viability Kit* was purchased from *Invitrogen* (Carlsbad, United States). Gentamicin was acquired from Shanghai Macklin Biochemical Co. Ltd (Shanghai, China). All other reagents were of analytical grade.

### Antibacterial Activity of Chlorogenic Acid on *Yersinia enterocolitica*

#### Poured-Plate Method

The diameters of the inhibition zone of CA against *Y. enterocolitica* were measured by the poured-plate method (Dai et al., 2018) with some modifications. Before starting the experiments, bacterial PBS (0.02 M, pH 7.4) suspensions with a concentration of 10^8^ CFU/mL were prepared. Briefly, 100 mL of sterilized LB agar (cool to 45°) was homogeneously mixed with 0.7 mL of diluted *Y. enterocolitica* to reach the final concentration of 10^4^ CFU/mL and then quickly poured into a sterile plate. After solidification, 6 holes with 8 mm diameters were made by sterile punch, and filled with 200 μL of sterile water, gentamicin (40 μg/mL), CA solutions (20, 40, 60, and 80 mg/mL CA), respectively. After incubation for 12 h in a 37° constant temperature incubator, the diameters of the inhibition zone were measured. Each experiment was performed in triplicate.

According to previous reports (Mitani et al., 2018), an acidic environment might contribute to the antibacterial effect to a certain degree. In order to exclude the possible effect of acidic characteristic of CA on its antibacterial function to *Y. enterocolitica*, the antibacterial effect of PBS with the same pH value as 80 mg/mL CA solution (pH 2.5) was assessed and compared with CA. The experiment was conducted as above.

#### Growth Curve

The time-dependent bactericidal kinetics of CA was analyzed by growth curve (Shi et al., 2018a). Bacterial PBS suspensions were obtained as above. Briefly, 0.5 mL of 20, 40, 60, 80, and 100 mg/mL CA were added into 4.5 mL of LB broth containing 10^6^ CFU/mL bacteria to make the final concentrations of 2, 4, 6, 8, and 10 mg/mL CA, respectively. All the samples were then incubated at 37° in a shaking incubator at 150 rpm. During the incubation, 200 μL of the culture was pipetted out to a sterile 96-well plate (*Corning*) at 2 h intervals to determine OD_600_ by a Multiskan FC Microplate Reader (*Thermo Fishe*r Scientific, United States). The same volumes of sterile water and gentamicin (40 μg/mL) instead of CA were used as negative and positive controls, respectively. The absorbance at each time point was used to plot a growth curve.

### Antibiofilm Activity of Chlorogenic Acid on *Yersinia enterocolitica*

#### Effect of Chlorogenic Acid on *Yersinia enterocolitica* Preformed Biofilm

To test the effect of CA on preformed biofilm of *Y. enterocolitica*, the biofilm of *Y. enterocolitica* was first formed and tested according to a previously reported method (Wang et al., 2020a) with some modifications. An overnight bacterial culture in LB broth was harvested and adjusted to 10^6^ CFU/mL using fresh LB broth. Aliquots of 200 μL bacterial suspension were added into a 96-well flat-bottomed polystyrene microtiter plate (*Corning*) and incubated for 24 h at 37° at the static condition to ensure the full adhesion of biofilm to the bottom of the plate. Following 24 h of bacterial adhesion and biofilm formation, the supernatants were decanted, planktonic cells were discarded, and the plate was rinsed three times with 200 μL PBS. Then, 200 μL 4, 6, 8, and 10 mg/mL CA and water were added into the wells, respectively, and incubated for 30 min at 37° with slight shaking. Afterward, the plate was washed three times using sterile water, fixed with methanol for 20 min, washed three times with water, stained with 0.1% (w/v) crystal violet for 10 min, and rinsed three times again. Finally, 200 μL of 95% ethanol was added to the crystal violet-stained wells, and the amount of biofilm formation was measured by the absorption at 595 nm using a Multiskan FC Microplate Reader (*Thermo Fishe*r Scientific, United States). The background from un-inoculated media was subtracted.

#### Inhibition of Biofilm Formation by Chlorogenic Acid

The inhibition of biofilm formation of *Y. enterocolitica* by CA was performed according to a previously reported method (Maury et al., 2019) with some modifications. An overnight bacterial culture in LB broth was harvested. Then, aliquots of 100 μL of the above prepared bacterial suspension were added into a 96-well plate (*Corning*) respectively, followed by the addition of 100 μL 8, 12, 16, and 20 mg/mL CA into the wells, respectively, ensuring the final bacterial concentration of 10^8^ CFU/mL in each well and the final concentration of 4, 6, 8 and 10 mg/mL CA. The plate was then incubated for 24 h at 37° at static condition, after which the remaining operation procedures were performed as mentioned above, and the absorbance of each well at 595 nm was obtained. The same volume of sterile water instead of CA was used as a negative control.

### Ultrastructure Observation

#### Scanning Electron Microscopy

The changes in microstructure and morphology of *Y. enterocolitica* after treatment with CA were imaged by Scanning electron microscopy (SEM) (Fang et al., 2018). Log-phase *Y. enterocolitica* with a concentration of 10^8^ CFU/mL was mixed with CA (final concentration was 10 mg/mL) and cultured for 30 min at 150 rpm at 37°. Sterile water was used as a control. The samples were treated according to the reference and finally observed via SEM (JSM-7500F, Hitachi, Japan).

#### Transmission Electron Microscopy

The intracellular alterations of *Y. enterocolitica* after treatment with CA were visualized by Transmission electron microscopy (TEM) (Li et al., 2019b). The treatment method of *Y. enterocolitica* with CA was identical to SEM protocol. The samples were treated according to the reference and finally observed via TEM (JEM-1200EX, JEOL Ltd., Tokyo, Japan).

### Bacterial Membrane Permeability

#### Propidium Iodide Staining

The Propidium iodide (PI) staining experiment was conducted using a previously reported method (Logue et al., 2018). Log-phase bacterial PBS suspensions were obtained as above. Specifically, *Y. enterocolitica* cells (10^8^ CFU/mL) were incubated with CA (final concentration was 10 mg/mL, 150 rpm, 37°) for 0.5, 3, 6, and 9 h, respectively. After incubation, bacteria were obtained by centrifugation at 6,000 rpm for 10 min, washed three times with 0.9% NaCl solution, re-suspended in NaCl solution, and diluted to approximate 10^6^CFU/mL. Then, 1 mL of the bacterial solution was mixed with 3 μL PI (20 mM in DMSO) in the dark for 15 min at room temperature. After that, the fluorescence intensity of samples was measured by a FACS AriaIII Flow cytometer (FC) (Becton Dickinson, Franklin Lakes, NJ, United States) at an excitation wavelength of 490 nm and an emission wavelength of 635 nm.

#### Laser Scanning Confocal Microscope

*LIVE/DEAD BacLight Bacterial Viability Kit* (*Invitrogen*, United States) was used to further elucidate the effect of CA on the cell membrane permeability (Watson et al., 2019). According to the specification, log-phase bacterial PBS suspensions (10^8^ CFU/mL) were incubated with CA (final concentration was 10 mg/mL, 150 rpm, at 37°) for 0.5, 3, 6, and 9 h, respectively. After incubation, bacteria were harvested by centrifugation at 6,000 rpm for 10 min, washed three times with 0.9% NaCl solution, resuspended in NaCl solution, and diluted to approximate 10^8^CFU/mL. Then, the bacterial solution was incubated with 3 μL mixed dye of PI: SYTO-9 (1:1) in the dark for 15 min at room temperature. After that, samples were dropped on a microscope slide and observed using a TCSsp5II Laser scanning confocal microscope (LSCM) (*Agilent*, United States) at the maximum excitation/emission wavelength 490/635 nm for PI and 480/500 nm for SYTO-9.

### Binding of Chlorogenic Acid to *Yersinia enterocolitica*

#### Quartz Crystal Microbalance

Quartz crystal microbalance (QCM) was used to test the binding of CA to *Y. enterocolitica*. Briefly, 50 μL of log-phase bacterial PBS suspension (10^8^ CFU/mL) was dropped on the surface of the Au chip and incubated for 24 h at 4°) at static condition to ensure the full adhesion of bacteria to the surface of the chip. Then, the chip was rinsed with slow water flow to remove the unattached bacteria and dried with nitrogen. Finally, the chip was placed into the sample room and analyzed by QCM (QE401-F1719, Q-sense, Biolin Scientific, AB, Finland). For the experiment, ultrapure water was first injected until the stable baseline. Then, 10 mg/mL CA and ultrapure water were sequentially injected until the baseline was stable. The equipment can examine the interaction through the frequency shifts [including resonance frequency (Δ*f*) and energy dissipation (Δ*D*)] of the electrode if CA could bind to *Y. enterocolitica*. A chip without bacteria attachment was used as a control to exclude the non-specific binding of CA to the chip.

#### Fluorescence Detection by Flow Cytometer

Chlorogenic acid can naturally emit fluorescence at 440 nm (using an excitation wavelength of 380 nm) (Wang et al., 2020b). In this study, the fluorescence detection method by FC was designed to test the binding of CA to *Y. enterocolitica*. Specifically, 200 μL of log-phase bacterial PBS suspension (10^8^ CFU/mL) was incubated with 400 μg/mL, 600 μg/mL, 800 μg/mL, 1 mg/mL, and 2 mg/mL CA for 30 min at 150 rpm, 37°), respectively. After incubation, the mixtures were centrifuged at 6,000 rpm for 10 min, washed three times with PBS to remove the unbound CA, and bacteria concentration was adjusted to approximately 10^6^ CFU/mL with PBS. Finally, the fluorescence intensity of samples was recorded by a FACS AriaIII flow cytometer (Becton Dickinson, Franklin Lakes, NJ, United States). The bacteria treated with ultrapure water instead of CA were used as a control.

### Transcriptomic Analysis

In order to investigate the effect of CA on bacterial physiological metabolism and deeply elucidate the antibacterial mechanism of CA, a transcriptome analysis was conducted. The log-phase bacterial PBS suspension was incubated with CA (final concentration was 10 mg/mL) for 30 min at 150 rpm, 37°. Then, bacteria were collected by centrifugation for 10 min at 6,000 rpm, 4°, followed by washing three times with PBS. Finally, the total RNA of *Y. enterocolitica* was extracted using TRIzol^®^ Reagent according to the manufacturer’s instructions (Invitrogen), and genomic DNA was removed using DNase I (TaKara). The RNA quality was determined by 2100 Bioanalyser (Agilent) and quantified using the ND-2000 (NanoDrop Technologies). The data generated from the Illumina platform were used for bioinformatics analysis. All the analyses were performed using the free online platform of Majorbio Cloud Platform^1^ from Shanghai Majorbio Bio-pharm Technology Co., Ltd. The same volume of sterile water instead of CA was used as a control, and each experiment was performed in triplicate.

A Per1 program was written to select clean reads by removing low-quality sequences (Q-value ≤ 20), reads with more than 5% of N bases (unknown bases), and reads containing adaptor sequences. For gene expression analysis, clean reads were mapped to reference using Bowtie2.^2^ The FPKM value was used to calculate the expression level. Differentially expressed genes (DEGs) among different samples were detected using DEseq2.^3^ An absolute fold change > 1.5 and p-value < 0.05 were set as the threshold to select the significant DEGs. Then, Gene Ontology (GO) and Kyoto Encyclopedia of Genes and Genomes (KEGG) analyses were performed to assign DEGs to different functional groups. Goatools^4^ and KOBAS 2.0 (see text footnote 4) were used to identify statistically enriched GO terms and enriched pathways using Fisher’s exact test, respectively. RNA-seq data for *Y. enterocolitica* were deposited in the NCBI Sequence Read Archive under accession number PRJNA812276.

In order to validate the gene expression results by RNA-seq, eleven genes were selected from *Y. enterocolitica* and quantified by qRT-PCR. All the primers for the real-time analysis are listed in Supplementary Table 1. The data were exported and quantified via the comparative Ct method (2^–Δ^ ^Δ^ *^Ct^*). All reactions were carried out in triplicate.

### Growth Inhibition Activity of Chlorogenic Acid on *Yersinia enterocolitica* in Milk

*Yersinia enterocolitica* growth inhibition was assessed in milk using a previously described approach (Tian et al., 2021) with some modifications. A 10 mL sterilized milk, purchased from a local supermarket, was firstly mixed with 100 μL of bacterial PBS suspensions at a concentration of 10^8^ CFU/mL, and then 100 mg CA was added into the above solution, yielding a final concentration of CA of 10 mg/mL. After incubation for 30 min at 150 rpm, 37°, the samples were diluted to a suitable concentration and spread on LB agar plate. Followed by incubating at 37° for 24 h, the bacterial counts were calculated. The same weight of sterile water instead of CA was used as a negative control. The growth inhibition rate was calculated according to the reference. Each experiment was done in triplicate.

### Statistical Analysis

All experiments were performed in triplicate. Data were presented as mean ± SD. One-way analysis of variance (ANOVA) was performed using SPSS 26 (SPSS Inc., United States). Statistical significance was considered at *P* < 0.05.

## Results

### Antibacterial Effect of Chlorogenic Acid on *Yersinia enterocolitica*

#### Poured-Plate Method

**FIGURE 1:**
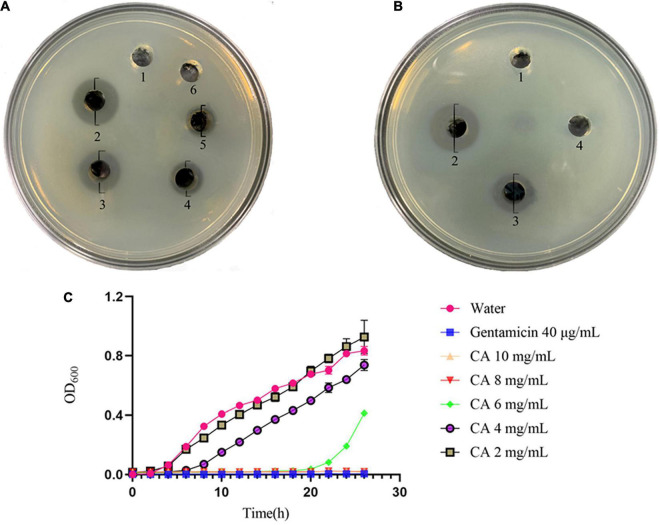
Antibacterial effect of CA against *Y. enterocolitica*. **(A)** Inhibition zones by water (1), 40 μg/mL gentamicin (2), 80 mg/mL CA (3), 60 mg/mL CA (4), 40 mg/mL CA (5), and 20 mg/mL CA (6). **(B)** Inhibition zones by water (1), 40 μg/mL gentamicin (2), 80 mg/mL CA (3), and PBS with pH of 2.5 (4). **(C)** Growth curves of *Y. enterocolitica* treated with water, gentamicin, and different concentrations of CA.

The *in vitro* antibacterial results of CA against *Y. enterocolitica* are shown in Figure 1A, and the diameters of the inhibition zone are shown in Supplementary Table 2. As seen in Figure 1A, CA exerted a dramatic growth inhibition on *Y. enterocolitica*. The diameters of the inhibition zone increased in a concentration-dependent manner ranging from 40 to 80 mg/mL, demonstrating that CA exerted antibacterial activity dose-dependently. Yet, no obvious inhibition zone was observed at 20 mg/mL CA in comparison with the gentamicin group.

Meanwhile, the effect of the acidic characteristic of CA on *Y. enterocolitica* was conducted (Figure 1B). The diameters of the inhibition zone are shown in Supplementary Table 3. Compared with 80 mg/mL CA, which exerted distinct antibacterial activity (with a diameter of inhibition zone of 13.5 mm), PBS with the same pH value as 80 mg/mL CA solution (pH 2.5) had no obvious antibacterial activity (no inhibition zone). Hence, it was concluded that the antibacterial effect of CA was not caused by its acidic characteristic but by other mechanisms of action.

#### Growth Curve

The growth curve was plotted to further test the antibacterial effect of CA. According to Figure 1C, compared with the negative control group, a significant decline of OD_600_ was observed in CA groups. At CA concentrations of 2, 4, and 6 mg/mL, the growth of *Y. enterocolitica* was initially repressed, and such a tendency lasted for 4-8 h, indicating the occurrence of partial bacteria inhibition or lysis by CA. However, at 8 mg/mL and 10 mg/mL CA, the growth curves were horizontal, and the OD_600_ values remained unchanged throughout the overall process, just as that of the positive control of gentamicin, indicating the complete bacterial inhibition or lysis by CA. Moreover, from the overall tendencies of growth curves, we concluded that the antibacterial activity of CA against *Y. enterocolitica* was dose- and time-dependent.

### Antibiofilm Activity of Chlorogenic Acid on *Yersinia enterocolitica*

**FIGURE 2:**
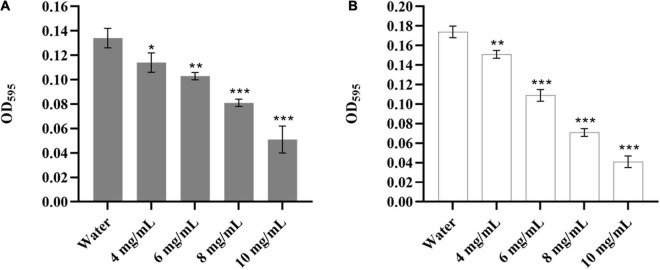
The biofilm susceptibility of *Y. enterocolitica* affected by CA. **(A)** The effects of different concentrations of CA on established biofilm of *Y. enterocolitica*. **(B)** The effects of different concentrations of CA on biofilm formation of *Y. enterocolitica*.

It was reported that *Y. enterocolitica* could form biofilms, which might be related to its pathogenicity, antibiotic resistance, and virulence (Ioannidis et al., 2014; Di Marco et al., 2020). Therefore, it was necessary to evaluate the effect of CA on the biofilm of *Y. enterocolitica*. As shown in Figure 2A, CA could significantly decrease the biomass of the established biofilm of *Y. enterocolitica* in a dose-dependent manner. Moreover, at 10 mg/mL, the reduction rate reached 61.9% compared with the control. Also, CA could significantly inhibit biofilm formation in a dose-dependent manner. Furthermore, at 10 mg/mL, the inhibition rate reached 76.4% (Figure 2B). This data suggests that CA could not only decrease the biomass of established biofilm but also inhibit the formation of biofilm of *Y. enterocolitica*.

### Ultrastructure Changes of *Yersinia enterocolitica* After Chlorogenic Acid Treatment

#### Scanning Electron Microscopy

**FIGURE 3:**
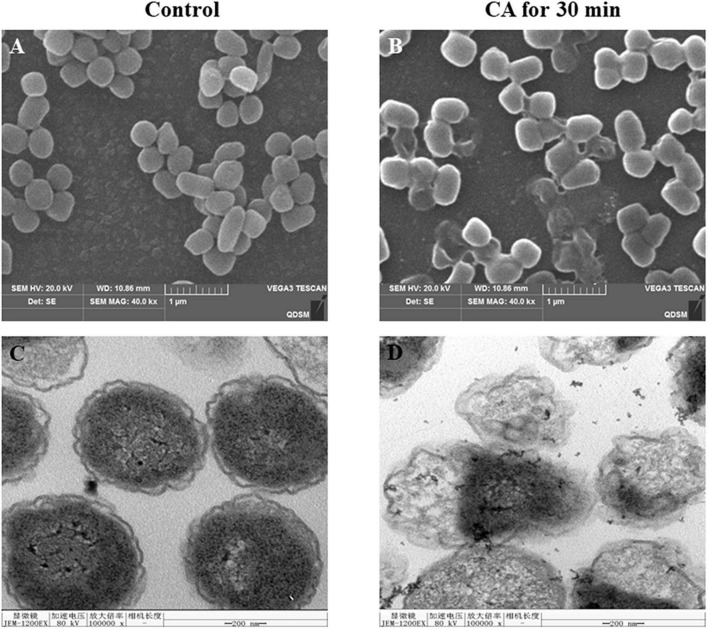
**(A,B)** Scanning electron microscopy (SEM) and **(C,D)** Transmission electron microscopy (TEM) images of *Y. enterocolitica* before and after treatment with CA.

The SEM microstructural analysis of *Y. enterocolitica* is shown in Figure 3A. In the control group, normal *Y. enterocolitica* was a typical rod-shaped structure with a smooth surface, and the average diameter was about 1 μm. However, after CA treatment, the shape of bacteria became irregular and wrinkled, and the surface of bacteria became rough. Moreover, some severe damages and ruptures of bacteria were observed, and the integrity of bacteria was destroyed.

#### Transmission Electron Microscopy

The destruction of the bacterial structure by CA was further observed by TEM (Figure 3B). The untreated bacteria were bacilliform and intact. However, after incubation with CA, the cell wall disappeared locally, and the bacterial integrity was ruptured. Moreover, the contents of bacteria flowed out, and the interior structure of bacteria was completely damaged.

### Effect of Chlorogenic Acid on Bacterial Membrane Permeability

#### Propidium Iodide Staining

**FIGURE 4:**
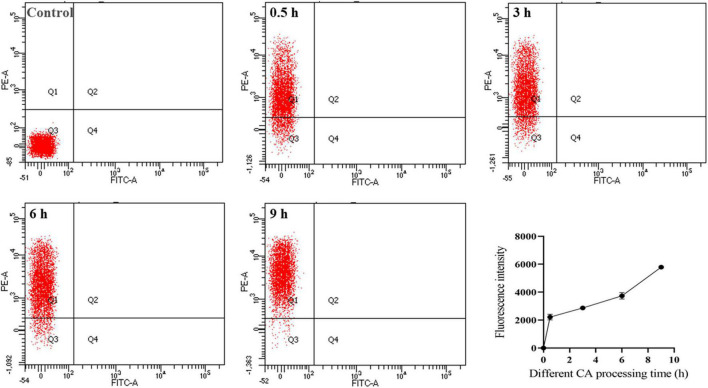
Flow cytometry plots of *Y. enterocolitica* after CA treatment.

To further demonstrate the antibacterial mechanism of CA against *Y. enterocolitica*, the bacteria treated with CA for a different time were stained with PI. As a kind of nucleic acid dye, PI can penetrate cells and bind to nucleic acid only when the cell membrane is disrupted. Thus, when the cell membrane was ruptured, PI entered the cell, and its fluorescence was reserved and could be detected by FC (Shi et al., 2018b). In short, the higher the fluorescence intensity, the more severe damage on the membrane. As shown in Figure 4, the fluorescence intensity of the bacteria after treatment with CA was significantly higher than that of negative control and increased with incubation time, indicating that CA could exert the bactericidal activity by disrupting the bacterial cell membrane, which was in accordance with the results of SEM and TEM.

#### Bacterial Membrane Permeability Studied by Laser Scanning Confocal Microscope

To further investigate if CA could cause changes in the bacterial cell membrane permeability, a live/dead cell staining method was performed using two nucleic acid dyes of SYTO-9 and PI. When SYTO-9 is used alone, all bacteria are stained (those with intact membrane and those with damaged membrane). On the contrary, PI can only penetrate the damaged bacteria (Shi et al., 2018b). However, when both dyes are used together, PI can conceal the fluorescent color of SYTO-9. Thus, red fluorescence can be seen in damaged bacteria. In other words, the higher red fluorescence intensity reflects the higher membrane permeability.

**FIGURE 5:**
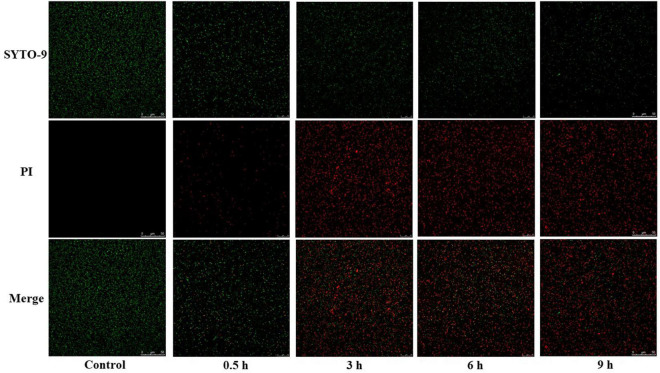
LSCM images of *Y. enterocolitica* after CA treatment.

As seen in Figure 5, bacteria with red fluorescence were observed after the treatment of CA, indicating that CA could increase the bacterial cell membrane permeability. Furthermore, with the extension of CA processing time, more bacteria with red fluorescence were observed, and bacteria with green fluorescence accordingly decreased. These results suggest that CA destroys the cell membrane of *Y. enterocolitica* and increases the permeability of the cell membrane in a time-dependent manner.

### Binding of Chlorogenic Acid to *Yersinia enterocolitica*

#### Quartz Crystal Microbalance

**FIGURE 6:**
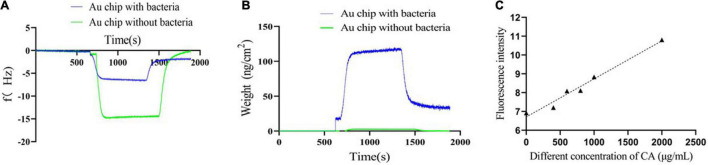
Verification of the binding of CA to *Y. enterocolitica*. **(A)** The frequency changes during binding. **(B)** The weight changes during binding. **(C)** The fluorescence intensities of *Y. enterocolitica* after treatment with different concentrations of CA.

Quartz crystal microbalance is a biosensor platform that operates on the principle of mass detection due to the piezoelectric effect. When a mass is adsorbed on the surface of the QCM electrode, the oscillation frequency of the electrode shifts and the weight changes (Speight and Cooper, 2012; Liu et al., 2013; Yilmaz et al., 2015; Emir Diltemiz et al., 2017; Feng et al., 2017). When the CA solution flowed through the Au chip surface with *Y. enterocolitica*, the frequency drastically increased until reaching a stable plateau. After using ultrapure water to rinse away the non-specific interaction, the frequency drastically decreased until reaching another one stable plateau, which was under the zero baseline (with the *f* value of *-*1.7912 Hz) (Figure 6A). Similarly, the weight changes on the Au chip had the same tendency line as *f* changes (with the weight of 32.05 ng/cm^2^) (Figure 6B). On the contrary, for the negative control, when the CA solution flowed through the Au chip without bacteria attaching to, the frequency also drastically increased until reaching stable plateau; but after rinsing with ultrapure water, the frequency drastically decreased until reaching the zero baseline (Figure 6A), suggesting no interaction. Differently, there were almost no weight changes for the negative control over the whole process (Figure 6B). The results indicated that the changes of *f* and weight were on account of the possible interaction of CA with *Y. enterocolitica*, and not because of the non-specific binding. Therefore, it can be inferred that specific binding of CA to *Y. enterocolitica* occurred.

#### Fluorescence Detection by Flow Cytometer

Based on the characteristic of CA, namely, emitting fluorescence at 440 nm at an excitation wavelength of 380 nm (Supplementary Figure 1), a fluorescence detection assay by FC was performed to further analyze the binding of CA to *Y. enterocolitica*. As shown in Figure 6C, after incubation with CA and removal of unbound CA, the fluorescence at 440 nm was detected by FC, indicating the binding of CA to *Y. enterocolitica*. In addition, the fluorescence intensities had a linear relation and a positive correlation with the concentrations of CA.

### Transcriptomic Analysis of *Yersinia enterocolitica* After Chlorogenic Acid Treatment

**FIGURE 7:**
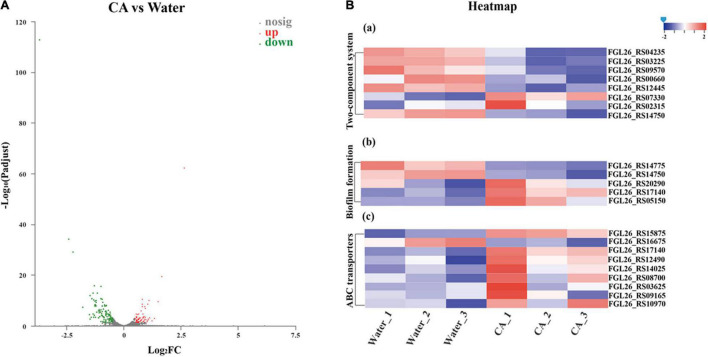
Transcriptome analysis of *Y. enterocolitica* after treatment with CA. **(A)** Volcano plot of expression of genes in *Y. enterocolitica* after treatment with CA. **(B)** Analysis of DEGs in *Y. enterocolitica* after treatment with CA. (a) Two-component system. (b) Biofilm formation. (c) ABC transporters.

Strand-specific prokaryotic transcriptome sequencing was performed to further reveal the potential antibacterial mechanism of CA against *Y. enterocolitica* from the perspective of changes in gene expression. In total, 154.9 million clean reads were obtained for *Y. enterocolitica*, and over 98.86% of clean reads were mapped to the reference genome sequences (Supplementary Table 4). Correlation analysis and principal component analysis (PCA) showed good biological duplications in our data and apparent differences between control and treatment groups (Supplementary Figures 2, 3). Counts of expression genes (FPKM > 1) identified 4,055 genes expressed in *Y. enterocolitica*. Among those genes, there were 179 DEGs in CA-treated *Y. enterocolitica* compared to the control group, with 69 genes being significantly up-regulated and 110 genes significantly down-regulated (Figure 7A and Supplementary Figure 4).

Based on the DEGs, GO and KEGG enrichment analyses were performed to obtain a comprehensive explanation (Supplementary Figures 5, 6). A total of 229 GO terms, including 81 (35.37%) molecular function terms, 22 (9.61%) cellular component terms, and 126 (55.75%) biological process terms, were assigned to 179 DEGs in *Y. enterocolitica*. Among the GO terms, 19 molecular function terms, 20 cellular component terms, and 87 biological process terms were significantly enriched (corrected *p*-value < 0.05). The top 20 enriched GO terms are shown in Supplementary Figure 5. Among the 20 enriched GO terms, 9 GO terms were related to the biological process, 9 GO terms were related to cellular components, and 2 GO terms were related to molecular function. In KEGG enrichment analysis, DEGs were allocated to 68 pathways. The top 20 enriched KEGG pathways are seen in Supplementary Figure 6. Among the top 20 enriched pathways, ribosome-related pathways had the most significant difference. 11 pathways were related to metabolism, 5 pathways were associated with human diseases, and 2 pathways were concerned with cellular processes. Moreover, both genetic information processing and environmental information processing involved 1 pathway.

In KEGG enrichment analysis, among these 179 DEGs, some important DEGs were allocated to pathways associated with biofilm formation (5 DEGs), membrane permeability (8 DEGs), ABC transporters (8 DEGs), and substance metabolism (70 DEGs), which might involve the growth inhibition or death of *Y. enterocolitica.* Five important DEGs were involved in biofilm formation-related pathways (Figure 7B). Specifically, an important gene, *luxS*, was up-regulated in two pathways associated with bacterial biofilms formation. Quorum sensing (QS) system, mediated by auto-inducers, is a cell-to-cell communication mechanism that regulates the expression of genes, and S-Ribosylhomocysteinase (LuxS)/autoinducer-2 (AI-2) system is an important member of QS system, which controls the expression of biofilm formation, virulence, and drug resistance (Yadav et al., 2018; Wang et al., 2019c). However, the up-regulated expression of *luxS* (FGL26_RS20290) was different from that reported in a previous study (Shi et al., 2018b). According to the explanation of Guan et al. (2021), the up-regulation of *luxS* expression could be explained by a protective response of *Y. enterocolitica*. *EnvZ* and *ompR* are critical parts in the bacterial Two-Component Regulatory System that mediate osmotic stress response and control the growth, metabolism, and mobility of bacteria (Tipton and Rather, 2017). In “Biofilm Formation” pathway, the expression of *envZ* (FGL26_RS14750) was significantly suppressed, which could decrease the biofilm formation of *Y. enterocolitica.* Moreover, *glgC*, an important gene that has a fundamental role in glycogen biosynthesis (Wang et al., 2020c), was down-regulated in *Y. enterocolitica* treated with CA. It is well known that the accumulation of glycogen in bacteria facilitates bacterial growth, increases the formation of bacterial biofilm, and improves tolerance to environmental stress (Klotz, 2017; Wang et al., 2020d). So, the downregulation of *glgC* (FGL26_RS14775) might inhibit the growth and biofilm formation of *Y. enterocolitica.* The gene *fumC* can encode fumarase, catalyzing the fumarate into malate. The previous report proved that elevated fumarate could promote the formation of *in vitro* biofilm of bacteria (Gabryszewski et al., 2019). Herein, the expression of *fumC* (FGL26_RS05150) was significantly increased, which could accelerate the conversion of fumarate and, in turn, decrease the formation of bacterial biofilm. To sum it up, CA could affect the biofilm formation of *Y. enterocolitica* by regulating the expression of a panel of genes related to biofilm formation, which was consistent with the results of the biofilm susceptibility assays above.

The Two-Component System acts as a key sensory pathway, enabling bacteria to sense, respond, and adapt to a wide range of environments and stressors. In this study, eight DEGs were involved in Two-Component System-related pathways (Figure 7B). Of those, three important genes were associated with bacterial growth, namely *ompF* (FGL26_RS07330), *kdpD* (FGL26_RS00660), and *cydB* (FGL26_RS03225). After treatment with CA, *ompF* was significantly up-regulated. OmpF is a β-barrel trimeric porin distributed on the outer membrane of Gram-negative bacteria, which acts as a major channel for the transport of nutrients and metabolites, and a major route for antibiotics to enter the bacteria (Lee et al., 2020). So, the up-regulation of *ompF* might induce the formation of larger holes on the outer membrane of *Y. enterocolitica*, causing an increase in cell membrane permeability, which was consistent with the membrane permeability assays above. The KdpD/KdpE system, an important Two-Component System, can sense chemical stimuli from the cytoplasm, especially K^+^ concentration, and conduct signaling transduction (Laermann et al., 2013). When histidine kinase KdpD perceives K^+^ limitation or salt stress, KdpD transfers the phosphoryl group to the response regulator KdpE and activates the high-affinity K^+^ uptake system KdpFABC (Heermann and Jung, 2010). On the contrary, the suppression of KdpD may inhibit the uptake of K^+^, which can destroy the intracellular homeostasis (Keppner et al., 2019) and affect the osmotic pressure (Sweet et al., 2020). Therefore, in this study, the down-regulation of *kdpD* might lead to the growth inhibition of *Y. enterocolitica* by this way. Furthermore, the KdpD/KdpE system has also been proven to exert influence on the expression of flagella-related genes and the flagella formation of avian pathogenic *E. coli* (Xue et al., 2020), which is essential for the motility and toxicity of bacteria (Yang et al., 2014), and has a critical role at the beginning of the biofilm formation of *B. cereus* and *Y. enterocolitica* (Di Marco et al., 2020). So, the down-regulation of *kdpD* might affect the biofilm formation of *Y. enterocolitica. CydB* is a part of the *cydAB* operon. It encodes cytochrome *bd* oxidase, which can prevent the accumulation of oxidative free radicals (Endley et al., 2001). Downregulation of *cydB* might result in inefficient ATP production (Takaoka et al., 2019) and reactive oxygen species increment (Endley et al., 2001). In this study, the gene *cydB* (FGL26_RS03225) was down-regulated in *Y. enterocolitica* treated with CA, which might result in adverse effects on bacterial growth.

ABC transporter systems catalyze the uptake of essential nutrients and the extrusion of toxic substances, thus acting as resistance factors against antibacterial peptides and antibiotics (Wilson, 2016). In this study, most of the ABC transporter systems associated DEGs were up-regulated (Figure 7B), indicating that the ABC transporter system might involve in the stress response of *Y. enterocolitica* exposed to CA.

qRT-PCR further suggested that the expression statuses of the selected genes were basically consistent with the trend of the RNA-seq results (Supplementary Figures 7A,B), indicating that RNA-seq was properly performed and validating the genetic evidence from transcriptional profiling.

### The Antibacterial Activity of Chlorogenic Acid on *Yersinia enterocolitica* in Milk

**FIGURE 8:**
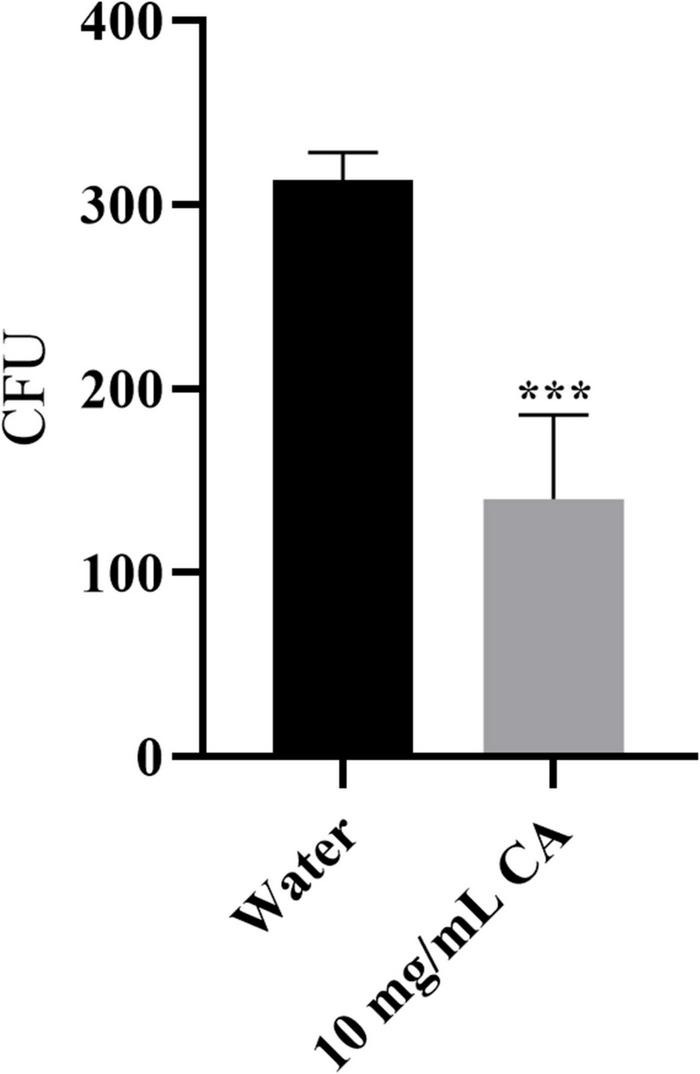
The growth inhibition effect of CA on *Y. enterocolitica* in milk.

*Yersinia enterocolitica*, as a gastrointestinal foodborne pathogenic bacterium, is commonly found in milk products (Savas and Altintas, 2019). Due to customers’ high food safety standards, the traditional chemical preservative used in milk is undesirable. Therefore, we assessed the antibacterial activity of CA on *Y. enterocolitica* in milk. As shown in Figure 8, CA could significantly decrease the amount of *Y. enterocolitica* by about 54.8% (*P* < 0.001), indicating the potential of application in milk preservation.

## Discussion

Developing new natural plant-derived compounds with outstanding antibacterial effects as alternatives or adjuvants of antibiotics has become increasingly popular over the years. In this study, the antibacterial effect of CA against *Y. enterocolitica* was investigated for the first time, and the mechanism of action was systematically illustrated.

The antibacterial activity of CA against *Y. enterocolitica* was verified by two commonly used methods, i.e., inhibition zone and growth curve experiments. Although the results of the two methods were consistent, the inhibitory concentrations of used CA were significantly different. In the inhibition zone experiment, only CA ≥ 40 mg/mL could generate a visible inhibition zone, while in the growth curve experiment, CA ≥ 8 mg/mL could completely inhibit the growth of *Y. enterocolitica*, which indicates that the growth curve method is more sensitive because of sufficient contact of the participants.

It has been proved that the formed biofilm of *Y. enterocolitica* strains could present enhanced antibiotic resistance (Ioannidis et al., 2014). Antibiotics mainly target planktonic cells rather than biofilms, while natural antibiotic compounds, like plant polyphenols, can inhibit bacterial adhesion and biofilm development (Stauder et al., 2010). Therefore, there is an urgent need for natural substances that inhibit both bacterial planktonic cells and biofilms. This study reported for the first time that CA could significantly reduce the adhesion of *Y. enterocolitica* to polystyrene (Figure 2A) and inhibit the formation of biofilms (Figure 2B), suggesting that CA could weaken the virulence, adherence, and antibiotic resistance of *Y. enterocolitica* by affecting the biofilms.

The effects of CA on the structure and integrity of *Y. enterocolitica* were reflected through several assays. The SEM and TEM results illustrated that CA could damage the cell wall and cell membrane of *Y. enterocolitica* and rupture the bacteria integrity (Figure 3). Moreover, the membrane permeability changes were tested by both FC and LSCM. The fluorescence of the dye PI was detected in the bacterial cells treated with CA by FC or LSCM (Figures 4, 5), indicating the damage of cell membrane and the increase of the permeability of cell membrane, which was consistent with the results of SEM and TEM.

Based on our results, we speculated the damage on *Y. enterocolitica* by CA was achieved through the binding of CA to the bacterial cell. To confirm the inference, two innovative experiments were performed, i.e., *f* and weight changes detected by QCM and fluorescence changes detected by FC. QCM is an excellent sensor to detect any interaction because of its ability to detect the oscillation frequency shifts of the electrode and weight changes when a mass is adsorbed on the surface of the QCM electrode (Feng et al., 2017). In this study, obvious *f* and weight changes were detected by QCM (Figures 6A,B), indicating the binding of CA to *Y. enterocolitica*, and proving that QCM could be used as a good tool to detect the interaction of two targets. On the other hand, the binding of CA to *Y. enterocolitica* was also confirmed by detecting the fluorescence of the bacterial cells treated with CA because of the characteristic of CA of emitting fluorescence at an excitation wavelength of 380 nm (Figure 6C). So, the binding is the first step for exerting its antibacterial effects. According to Francisco et al. (2013), CA has strong polarity and a high affinity for macromolecular substances such as lipids. It could bind to the surface of bacteria and change their membrane structure, increasing the permeability of the bacterial membrane, causing the leakage of intracellular components, and affecting protein synthesis. However, further studies are needed to determine what component of the cell membrane CA binds to, how the binding functions, and if the binding affects other cellular metabolisms.

**FIGURE 9:**
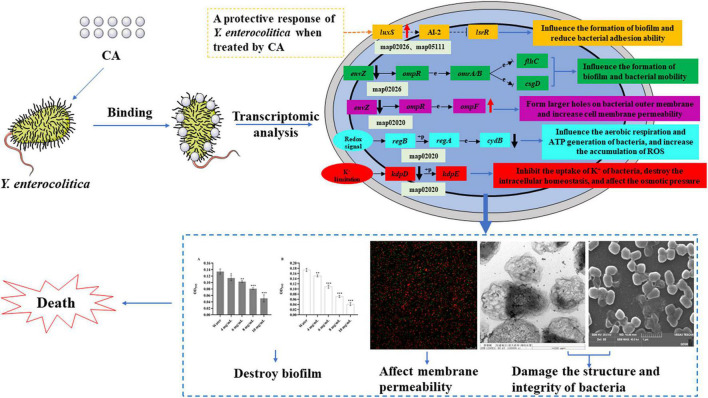
The scheme of antibacterial mechanism of CA against *Y. enterocolitica*.

Phenotypic changes are usually induced by differential gene expression. Therefore, to further investigate the antibacterial mechanism of CA on *Y. enterocolitica*, the transcriptome analysis was conducted, and the antibacterial mechanism was also comprehensively discussed by combining all the results (Figure 9). The transcriptomic results showed that CA affected a lot of physiological and metabolic pathways (Figure 7), indicating the negative effects of CA on the growth of *Y. enterocolitica*. Therefore, CA plays the antibacterial role on *Y. enterocolitica* through multiple ways. Two-component systems and biofilm formation associated pathways have a key role in the damage or apoptosis of *Y. enterocolitica* because of the abnormal expression of many important genes such as *luxS*, *glgC*, *envZ*, *ompF, kdpD*, *cydB*, etc., leading to the increase in permeability and the damage of biofilm, which was consistent with the results of membrane permeability and biofilm susceptibility assays above. Combined with all the assays we performed, it could be concluded that CA exerts antibacterial activity against *Y. enterocolitica* through intervening multi-physiological pathways, mainly biofilm formation and membrane permeability-related pathways.

As a natural antibacterial agent, it is necessary to test its performance in a food matrix which may introduce many disturbances. Herein, milk spiked with *Y. enterocolitica* was prepared, revealing that CA could significantly inhibit the growth of *Y. enterocolitica* in milk, and indicating the potential of application in milk preservation.

## Conclusion

In this study, it was proved for the first time that CA possessed significant antibacterial effects on *Y. enterocolitica*, which further broadened the antibacterial spectra of CA. Regarding the mechanism of action, it was demonstrated that CA could destroy the cell membrane and structure integrity, increase the membrane permeability through binding to *Y. enterocolitica*, causing the leakage of intracellular components, and finally leading to the cell death. Moreover, CA had outstanding anti-biofilm effects, including significantly decreasing the established biofilms and inhibiting the formation of biofilms, suggesting that CA could weaken the virulence, adherence, and antibiotic resistance of *Y. enterocolitica* by affecting the biofilms. Satisfactorily, transcriptomics analysis gave consistent results. Meanwhile, the growth of *Y. enterocolitica* in milk was significantly inhibited by CA, indicating the application potential in milk preservation. Taken together, CA, as an effective bactericidal effector, exerts antagonistic activity against *Y. enterocolitica* by mainly intervening biofilm formation and membrane permeability-related physiological pathways. But what component of *Y. enterocolitica* that CA binds to and how the binding functions need to be further studied. In a word, this study will be helpful to promote the application of CA, and even to tackle the tough problems of antibiotic resistance.

## Data Availability Statement

The datasets presented in this study can be found in online repositories. The names of the repository/repositories and accession number(s) can be found below: NCBI SRA - SRR18212738–SRR18212743.

## Author Contributions

KC: conceptualization, methodology, investigation, and writing – original draft. CP: methodology and writing – review and editing. FC: methodology and investigation. CY: writing – review and writing. QY: conceptualization, supervision, and writing – review and editing. ZL: writing – review and editing. All authors contributed to the article and approved the submitted version.

## Conflict of Interest

The authors declare that the research was conducted in the absence of any commercial or financial relationships that could be construed as a potential conflict of interest.

## Publisher’s Note

All claims expressed in this article are solely those of the authors and do not necessarily represent those of their affiliated organizations, or those of the publisher, the editors and the reviewers. Any product that may be evaluated in this article, or claim that may be made by its manufacturer, is not guaranteed or endorsed by the publisher.

## Footnotes

1
www.majorbio.com

2
http://bowtie-bio.sourceforge.net/bowtie2/index.shtml

3
http://bioconductor.org/packages/release/bioc/html/DESeq2.htmh

4
https://github.com/tanghaibao/GOatools
