# Supplementary material for: Antibacterial and Antibiofilm Activities of Chlorogenic Acid Against Yersinia enterocolitica
Source: Front Microbiol. 2022 May 4;13:885092. doi: 10.3389/fmicb.2022.885092 (PMC9117966; doi:10.3389/fmicb.2022.885092)
Supplement: Supplementary file 1 [file Data_Sheet_1.docx]

***Supplementary Material for***

Antibacterial and antibiofilm activities of chlorogenic acid against *Yesinia enterocolitica*

**Supplementary Table 1.** Sequence of primers for *Y. enterocolitica*.

| Genes | Forward primer | Reverse primer |
| --- | --- | --- |
| *16S rRNA* | GCACGTAATGGTGGGAACTC | CTCCAATCCGGACTACGACA |
| *ftnA* | TGACCACACACGATTACTCCACATTC | TTACCCACCAATGCCAGTTTATCCAG |
| *envZ* | GCAGGTCGGTAAGGGCATCATTC | TGCGGTCATCAGCCAACAACTTC |
| *FGL26_RS06420* | GAGAACAACCTGATGAGCTACGAGAG | CATACGGCAAGCGAGAGAATTTATTGG |
| *sodA* | CACTGCCATCCCTGCCTTATGC | GGTTTGGTGGTGTTTGGTGTGATG |
| *fepB* | CGATATACCAGACAGAGCCGAATGC | TCATACAACTTCAACGCCGAATCTCC |
| *FGL26_RS18305* | CAACGCCTGTAACCTCGCTAAATAATG | TCTTTAAATGCCGGGTCCCAAATCC |
| *recN* | CTGTTACGCCAGTTGGGTGAGTC | AGTTTCAGTTTCAGTGCCATCAGTTTG |
| *FGL26_RS14635* | TACCAATATCACCACCGATGCCAATG | ACAATCAGAACCAGCAGCAGCAG |
| *ystA* | ACAGTTTCAGGGCAGTTCAGTGATG | CAACATACATCGCAGCAATCCCAATC |
| *FGL26_RS05950* | TGGCTGGCTATTCGCAATCAAGG | CGCTATTGGCAGACGCAGGAAC |
| *tpx* | CGTGCTGTCGTGATTCTGGATGG | TCATAGTCTGGTTCAGTGGTGATTTCG |

**Supplementary Table 2.** The dimeters of inhibition zone of different concentrations of CA against *Y. enterocolitica*.

| Solutions | The dimeters of inhibition zone (mm) |
| --- | --- |
| Water | - |
| 80 μg/mL Gentamicin | 15.5 ± 0.030 |
| 80 mg/mL CA | 14.3 ± 0.015 |
| 60 mg/mL CA | 12.4 ± 0.025 |
| 40 mg/mL CA | 11.8 ± 0.079 |
| 20 mg/mL CA | - |

“- “: No antibacterial effect

**Supplementary Table 3.** The dimeters of inhibition zone of CA and PBS against *Y. enterocolitica*.

| Solutions | The dimeters of inhibition zone (mm) |
| --- | --- |
| Water | - |
| 80 μg/mL Gentamicin | 14.8 ± 0.020 |
| 80 mg/mL CA | 13.5 ± 0.025 |
| PBS (pH=2.5) | - |

“- “: No antibacterial effect


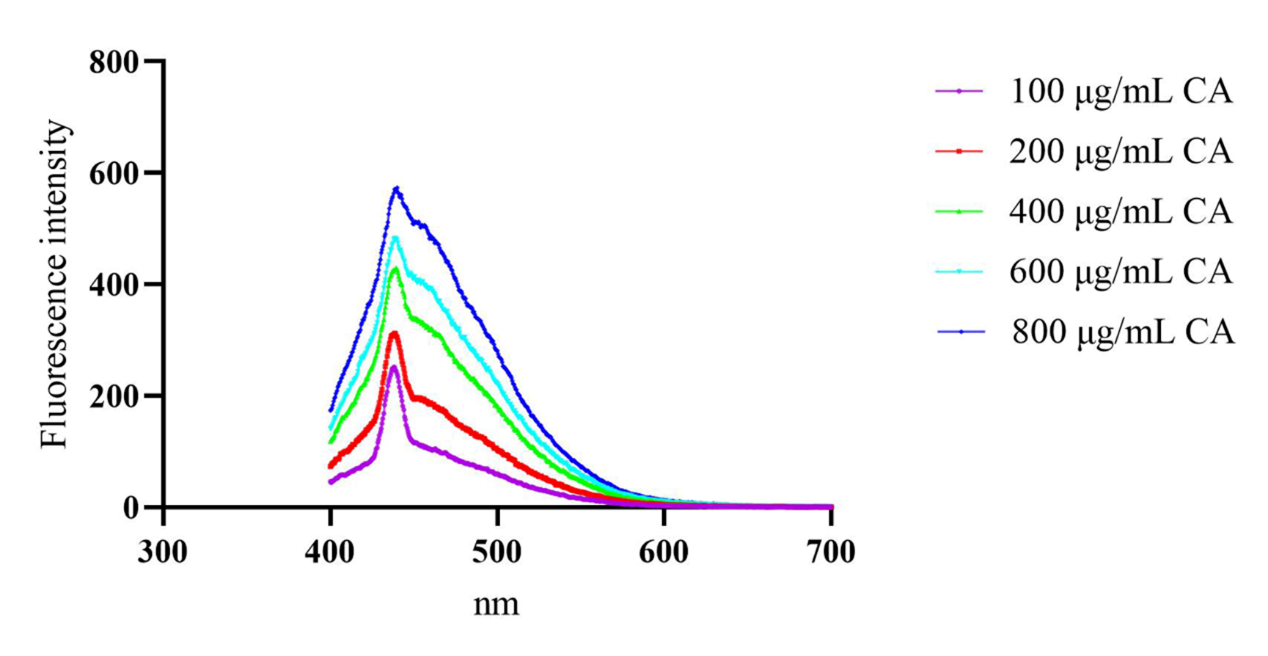


**Supplementary Figure 1.** The fluorescence emission spectrum of CA.

**Supplementary Table 4.** Summary of RNA-Seq data generated for *Y. enterocolitica* samples.

| **Sample name** | **Raw reads** | **Clean reads** | **Clean bases** | **Total mapped (%)** |
| --- | --- | --- | --- | --- |
| Water_1 | 23701606 | 23492938 | 3.22G | 98.86 |
| Water_2 | 24604996 | 24395678 | 3.34G | 98.95 |
| Water_3 | 27846512 | 27590116 | 3.72G | 98.92 |
| CA_1 | 25369094 | 25146788 | 3.44G | 98.87 |
| CA_2 | 26986356 | 26768840 | 3.64G | 98.93 |
| CA_3 | 26395978 | 26174368 | 3.58G | 98.87 |


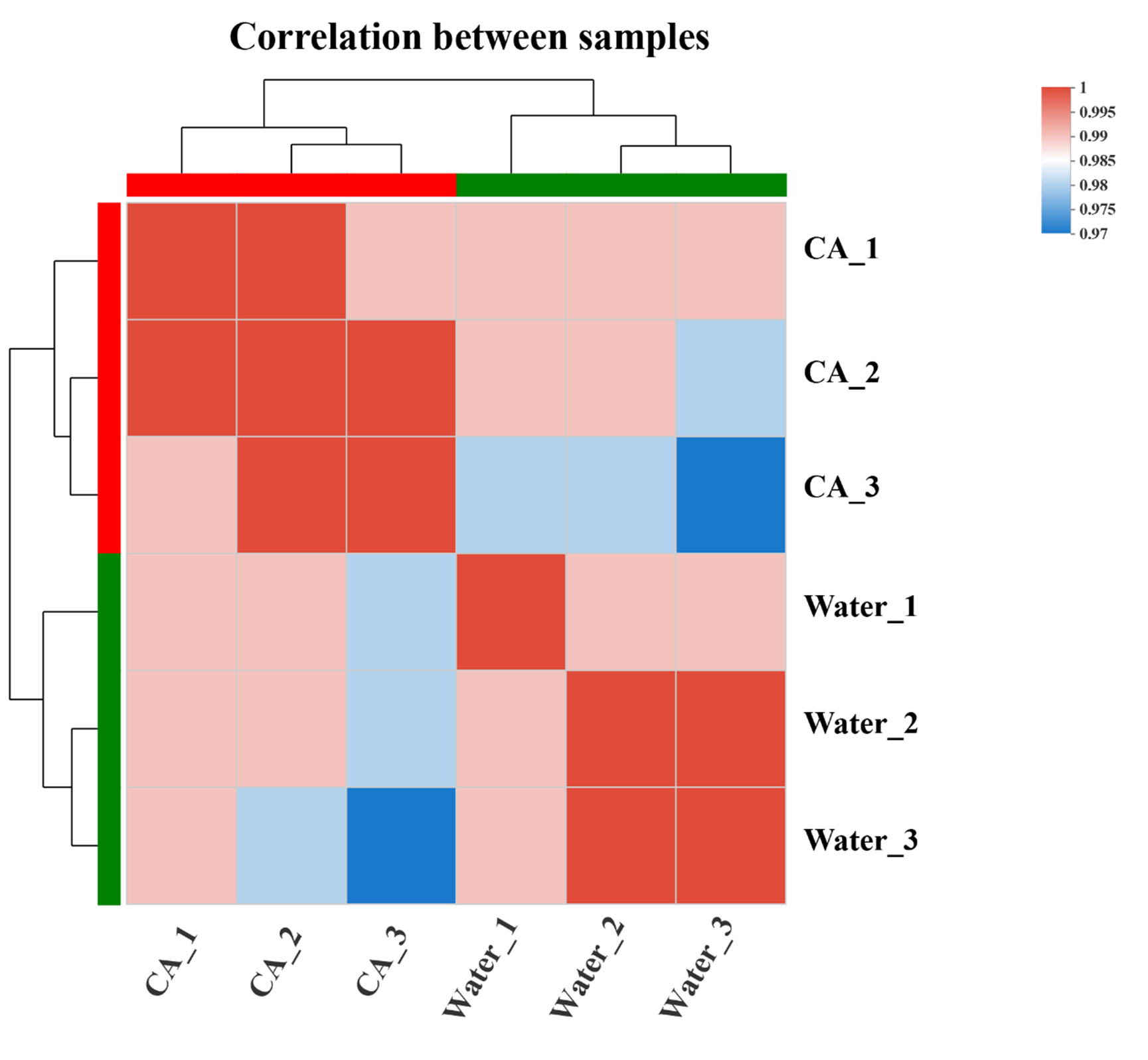


**Supplementary Figure 2.** Correlation analysis between *Y. enterocolitica* with and without CA treatment.


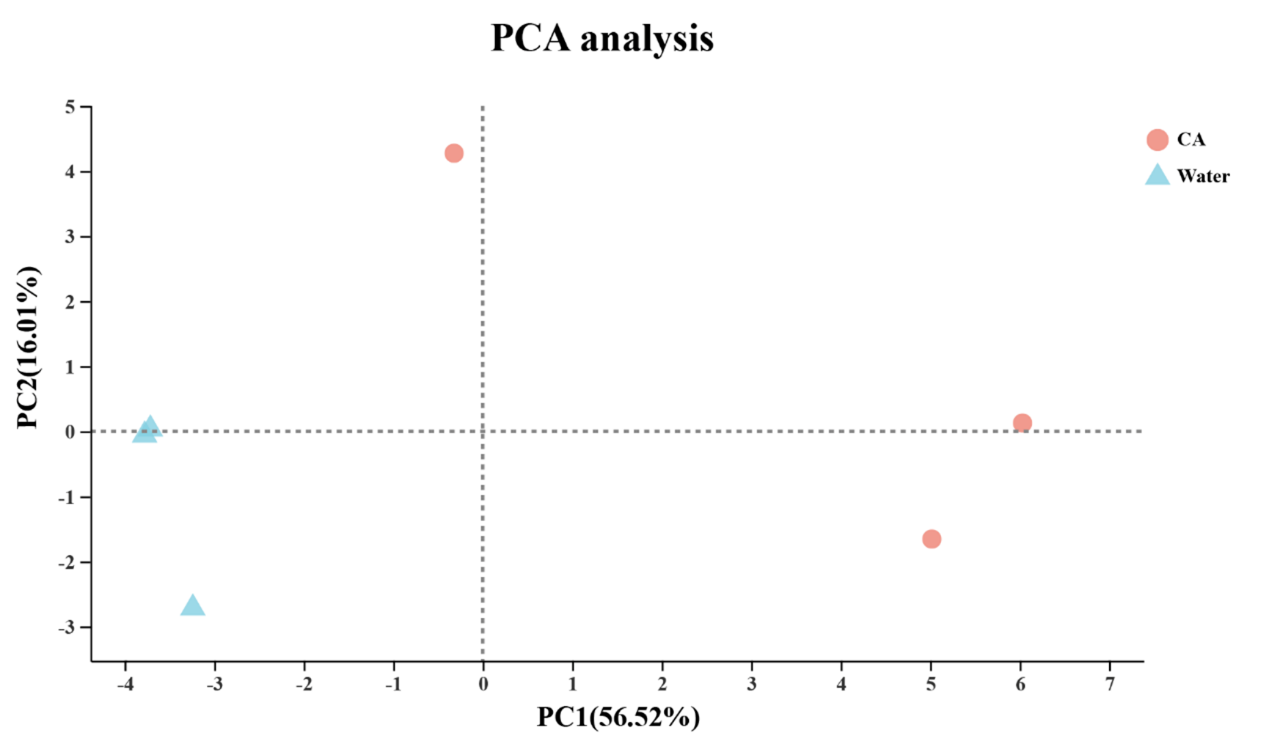


**Supplementary Figure 3.** PCA plot for *Y. enterocolitica* with or without CA treatment.


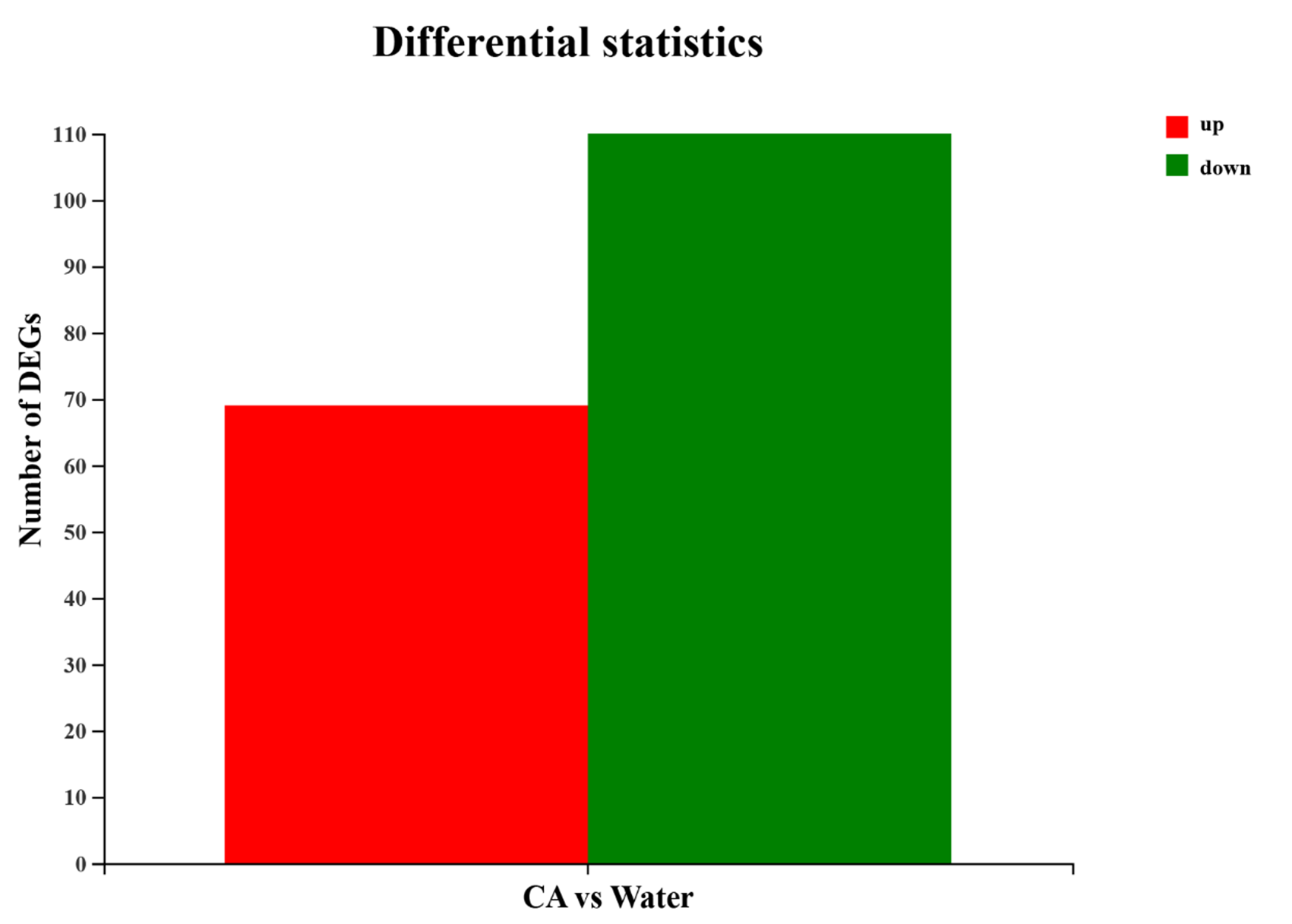


**Supplementary Figure 4.** Different expressed genes in *Y. enterocolitica* after treatment with CA.


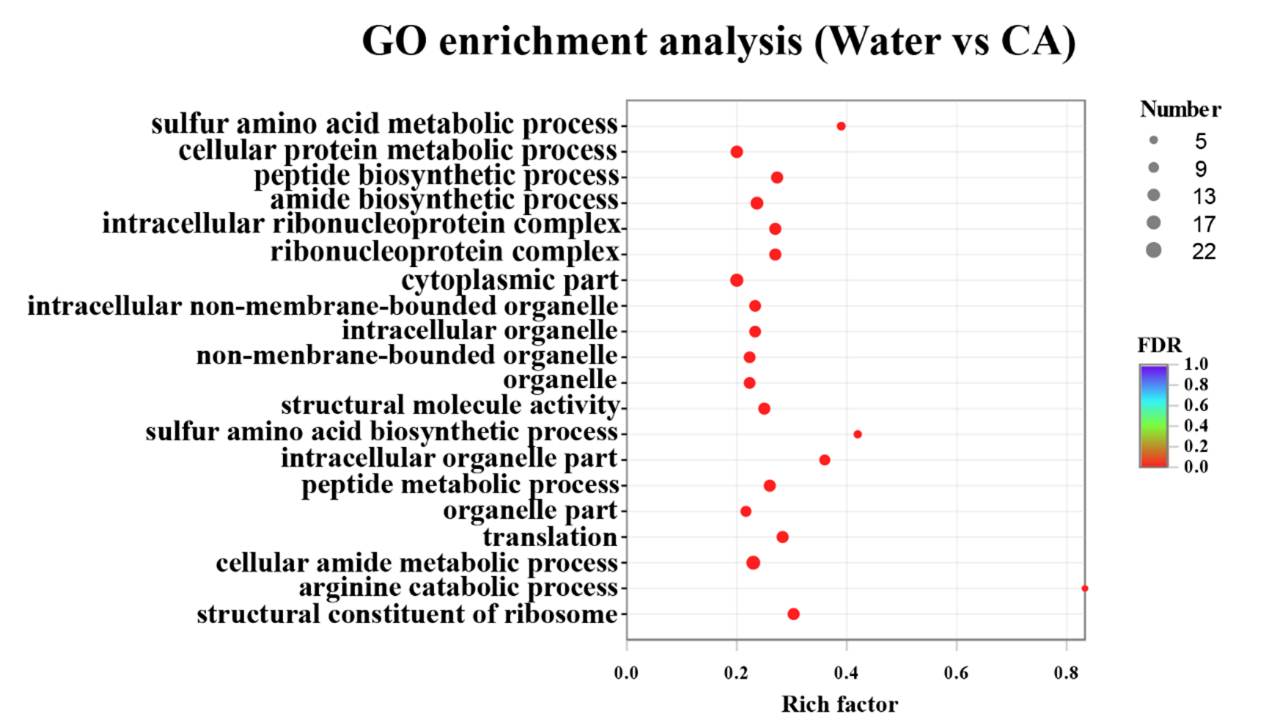


**Supplementary Figure 5.** GO enrichment analysis of DEGs in *Y. enterocolitic*a after CA treatment.


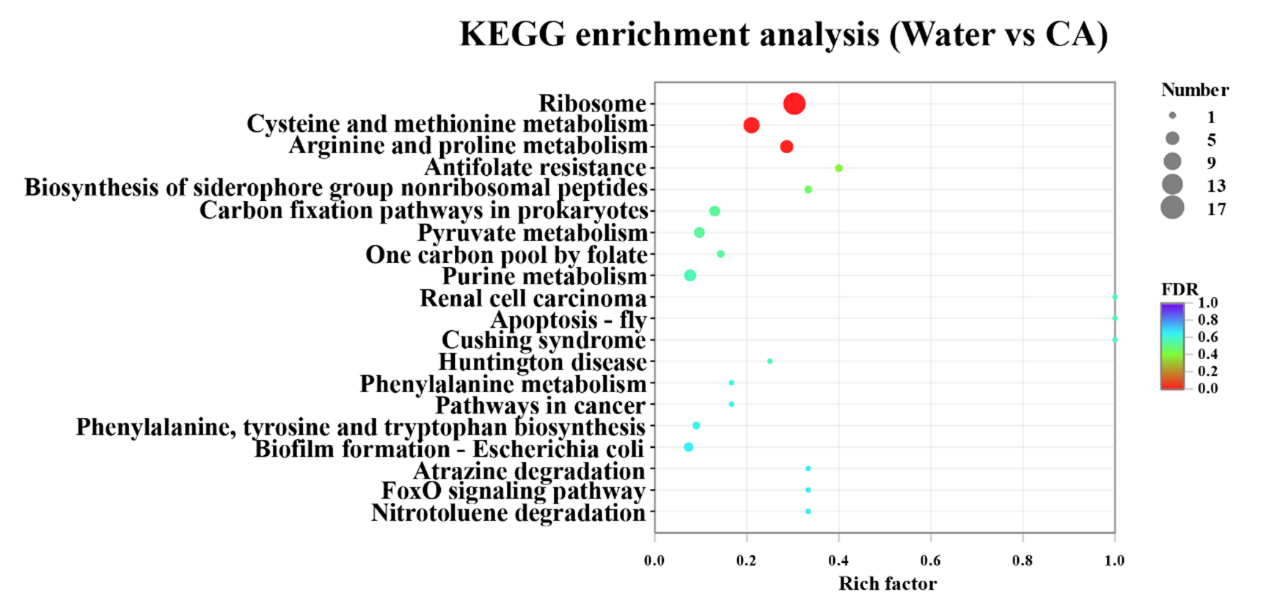


**Supplementary Figure 6.** KEGG enrichment analysis of total DEGs in *Y. enterocolitica* after CA treatment.


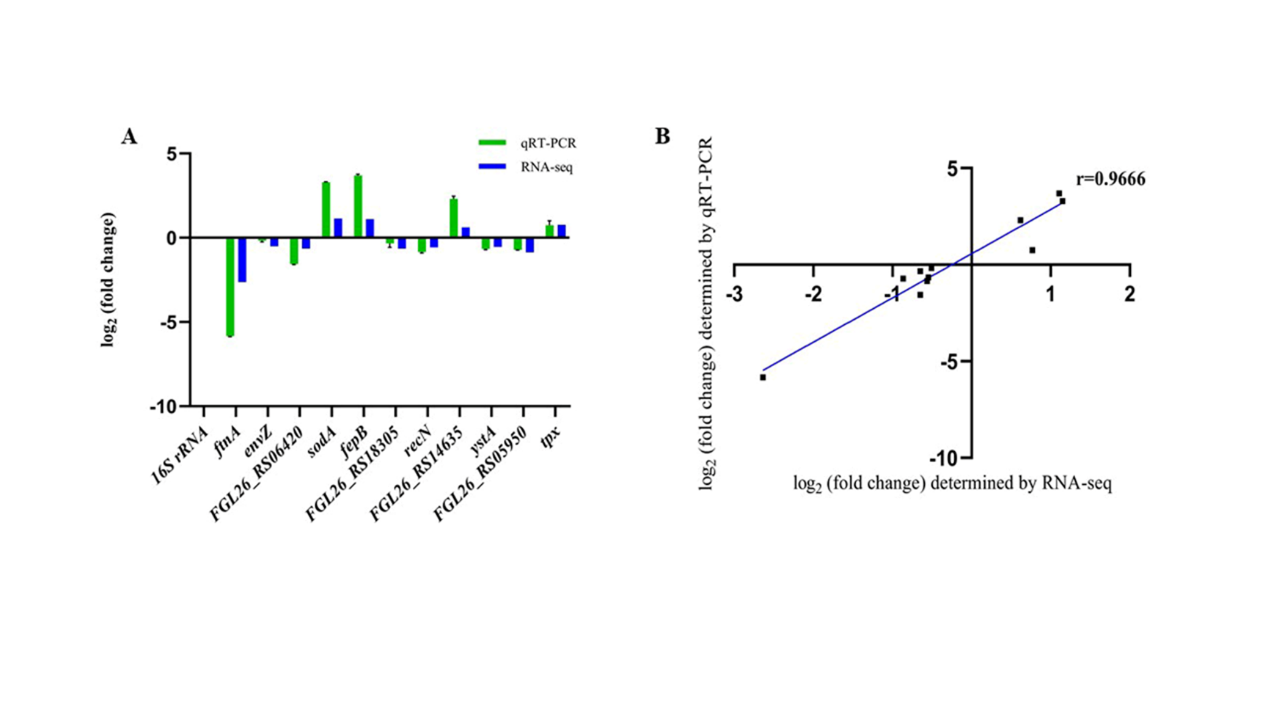


**Supplementary Figure 7.** qRT-PCR verification (A) and correlation analysis (B).
